# Extract

**Published:** 1886-09

**Authors:** 


### EXTRACT.

From an editorial in the *British Medical Journal*, of August 14th, relative to the meeting of the British Medical Association at Brighton, the following is taken: The proceedings were greatly heightened in interest by the attendance of a delegation from the International Medical Congress, to be held in America, at Washington, in September, 1887, to present an invitation to attend the Congress to the members of the British Medical association. There have been some considerable internal dissensions among the organizers of this Congress, but it was very generally felt that this ought not to and could not prevent the invitation from America from receiving a cordial acceptance. This invitation, which was made at once in the name of the International Medical Congress by its President-elect, and of the American Medical Association by some of its principal officers, was received with that sincere and affectionate cordiality which both the purport of the message and the sources from which it came already assured for it in advance. It lost nothing of dignity or of interest in the weighty and grave words of Dr. N. S. Davis, one of the oldest and most respected members of the profession in America, and one who holds very much the same relation to the American profession as Sir Charles Hastings long held to the profession in Great Britain. Dr. Davis was practically the founder of the American Medical Association, and is its most respected spokesman. The English profession may be sure that they will receive in America a welcome of unlimited hospitality, and of boundless kindness and brotherly affection. They will see there a great country and a great profession, which has sprung up and developed with a rapidity and to an extent which the world has never seen during the life time of many who were present to hear the invitation. In our own country, Paget, Lister, Mac Cormac; In France Charcot, Trèlat; and in all the countries of Europe, leading representatives of science have accepted invitations to be enrolled on the list of Vice Presidents. In America, the President of the United States, and the great officers of State, have signified their acceptance of the offices of Patrons of the meeting; and official recognition of the highest kind, such as is naturally due to a great gathering of medical men from all parts of the world, will be accorded to those who accept the invitation to attend the first great International Medical Congress which has ever been held in the United States.

### ABSTRACT.

CURABLE HYPERTROPHIC CERVICAL PACHYMENINGITIS. By Dr. Edgard Hertz. (*Archives Generales de Medecine, Juin,* 1886.)

Hertz briefly considers the somewhat meagre literature of the subject, and then reports two cases coming under his own observation. In addition, he details two cases reported by O. Berger (*Deut. Med. Wochenschrift,* 1878), one by Fere (*Progres Medical,* 1883), one by Joffroy (*Arch. Gen. de Med.,* 1876), and one by Burtin (*These de Paris,* 1878). From these observations he feels justified in formulating the following conclusions:

1. That the disease is caused in the majority of instances by exposure to cold, living in damp dwellings and prolonged exposure to currents of air.

2. That an analysis of the symptoms confirms the division of the disease into three periods: pain, paralysis and atrophy. For the latter we may advantageously substitute the term period of trophic disturbance, as in fact a veritable atrophy never exists, the muscles preserving the normal volume.

3. That there is a great excess in the quantity of urea excreted. In one case amounting to 76 grammes per litre. Improvement is accompanied by a fall in the proportions of urea in the urine.

4. That the disturbances of the sexual functions follow the same course, at first true satyriasis exists, followed by absolute but temporary impotency.

5. That the course of the disease justifies the title of the paper. Cervical pachymeningitis *is curable,* rarely in the first period of the disease, but during the period of paralysis and trophic changes.

6. That from the ob ervations of Joffroy, Féré and Burtin, sensible amelioration of the symptoms may be expected in about eighteen months. Prognosis is, however, unfavorable when the disease affects the lower extremities; when the bladder and rectum are affected, or when the deformity of *main en griffe* exists, with wasting of the muscles of the shoulder and the flexors of the fore-arm.

7. That the prognosis should be favorable in the largest number of cases, and that recovery does not take place progressively, but by sudden improvements followed by temporary relapses which necessitate continuous surveillance and moderate and prudent interference.

8. That the treatment in the painful period should consist in the employment of anodynes, chloral or morphine subcutaneously, and counter-irritants should be freely applied to the cervical region, particularly scarifications and the actual cautery. Prolonged hot baths are of great service. Electricity exercises a favorable influence over the trophic disturbances. The salicylate of soda is of great value in many cases, as the disease is frequently of rheumatic origin.

HAROLD N. MAYER.

